# Examining Attitudes and Experiences of Ageism During COVID-19: Are They on the Rise?

**DOI:** 10.1093/geroni/igab046.2302

**Published:** 2021-12-17

**Authors:** Michael Vale, Jennifer Sublett, Toni Bisconti

**Affiliations:** 1 Sacred Heart University , Trumbull, Connecticut, United States; 2 The University of Akron, Akron, Ohio, United States

## Abstract

Gerontologists have warned of rising ageism amidst the COVID-19 pandemic. Older adults have been portrayed as a homogenous group given their health vulnerabilities and have been viewed with mixed perceptions. For instance, the pandemic has been viewed as an “old person’s” disease and older adults have been inherently linked to imposing health and safety lifestyle changes. Others have responded with acts of overaccommodative care that have minimized older adults’ autonomy. Taken together, there have been inferences of increased hostile and benevolent ageism. Currently, these claims lack empirical data, and the goal of this study was to examine if attitudes and experiences of ageism are on the rise. Across two studies, we examined young adults’ (N=268) attitudes of older adults and older adults’ (N=65) experiences of ageism before and after the start of the pandemic. In study 1, we examined ageist attitudes at 3 time points (2017, 2019, 2020) from separate, but equitable, college samples and found that hostile ageism was higher during the pandemic (F(2,265)=5.48, p<.001) and benevolent ageism demonstrated no differences. In study 2, we explored older adults’ experiences of ageism pre-and post-pandemic onset (2019, 2020) and, found that they reported experiencing less hostile ageism (t(64)=2.45, p<.05), with no differences in experienced benevolent ageism. Our findings suggest an increase in hostile ageist views, but a decrease in experiences, partially supporting the alleged claims of rising ageism. Nevertheless, the last year of the pandemic is dynamically contextualized and research should elaborate on the extent and consequences of this rise.

